# Cultivar and Harvest Month Influence the Nutrient Content of *Opuntia* spp. Cactus Pear Cladode Mucilage Extracts

**DOI:** 10.3390/molecules23040916

**Published:** 2018-04-16

**Authors:** Alba du Toit, Maryna de Wit, Arno Hugo

**Affiliations:** 1 Department of Consumer Science, Faculty of Natural and Agricultural Sciences, University of the Free State, Bloemfontein 9300, South Africa; 2 Department of Microbial, Biochemical and Food Biotechnology, Faculty of Natural and Agricultural Sciences, University of the Free State, Bloemfontein 9300, South Africa; DeWitM@ufs.ac.za (M.d.W.); HugoA@ufs.ac.za (A.H.)

**Keywords:** prickly pear, nopal, *Opuntia ficus-indica*, *Opuntia robusta*, hydrocolloid gum, dietary fibre, calcium oxalate crystals, minerals and trace elements

## Abstract

Mucilage extracted from cactus pear cladodes is a hydrocolloid gum. It is a novel, natural, low-kilojoule, cost-effective texture-modifying ingredient in functional food products. Yet, the cultivar with the most optimal nutrient content and the preferred harvest times are as yet unknown. For this reason, mucilage from three *Opuntia ficus-indica* (Algerian, Morado and Gymno-Carpo) and one *Opuntia robusta* (Robusta) cultivar were investigated to determine their nutrient content over six months. Nutrients that contribute energy (10.2 kJ/g) were low. The mineral content was high (ash 17.7/100 g), particularly calcium (3.0 g/100 g) and phosphorous (109.5 mg/kg). Low insoluble acid-detergent fibre (1.4 g/kg) and neutral-detergent fibre (2.1 g/kg) values indicated that mucilage was mostly soluble fibre. Calcium oxalate crystals were not detected in dried mucilage. *Opuntia robusta* powders had higher protein, extractable fat and potassium content, while *Opuntia ficus-indica* mucilage powders had higher polyunsaturated (Linoleic and α-Linolenic acid) fat content. *O. robusta* Robusta mucilage, harvested after the fruit harvest (February) had the lowest energy content and the highest mineral and protein content. Mucilage powders were highly soluble, low-kilojoule and mineral-rich. This is a functional ingredient that is produced from an easily cultivated crop, as cactus pears grow in areas with poor soil, extremely high daytime temperatures and limited water supplies.

## 1. Introduction

Food developers, with health-conscious consumers in mind, are interested in low-kilojoule, natural, cost-effective and texture-enhancing hydrocolloids [1]. Hydrocolloids are used to modify textural properties of food when ingredients such as gluten, fats, egg and dairy products are omitted in order to develop low-kilojoule or allergy-free functional food products [2]. Cactus pear cladodes contain a slimy substance (mucilage) that is a hydrocolloid and exhibits promising functional and rheological properties [2,3].

Mucilaginous molecules found in cladodes are long-chain acidic hetero-polysaccharides, which increase viscosity when dissolved [4]. The extracted mucilage is a non-Newtonian liquid, able to absorb and hold huge amounts of water in its structure [4]. Previous research has demonstrated its unique rheological properties, the occurrence of pseudoplastic (shear-thinning) behaviour, and the influence of temperature, pH and ionic strength fluctuations during processing, preparation and consumption of food products containing mucilage [5].

The mucilage consists mostly of soluble fibre, which is an important component of a healthy diet [1,6]. Additionally, cactus pear products have been proven to be effective in the treatment of ailments and has numerous therapeutic properties [7,8]. The development of mucilage-containing functional food products would not only benefit consumers by providing natural remedies and treatments for chronic diseases such as cancer, diabetes mellitus and cardiovascular diseases, but would also develop the cactus pear as a commercial crop [7].

Cactus pear plants have to retain water at all costs, in addition to obtaining CO_2_, and are adapted to use crassulacean acid metabolism (CAM), which is an alternative to photosynthesis. The stomata are only open at night for CO_2_ fixation when water-loss is limited [9]. CAM is more efficient during long, sunny summer days; thus, the cactus pear thrives in summer and struggles in cold frosty winters. The commercial cultivation and research of *Opuntia ficus-indica* and *Opuntia robusta* in South Africa is limited to the spineless Burbank varieties, which are susceptible to frost [10].

Of the cactus pear cultivars available for research, four were feasible for the commercial production of mucilage powders. These cultivars included *O. ficus-indica* cv Algerian (pink fruit) with low-viscosity and high-yield mucilage, *O. ficus-indica* cv Morado (green fruit) with high viscosity, yield and commercial fruit production use, *O. ficus-indica* cv Gymno-Carpo (orange fruit) with medium-high mucilage viscosity and yield and *O. robusta* Robusta (purple fruit), which is well known as animal fodder.

The nutrient content of freeze-dried mucilage may be influenced by cultivar, species, growth stage and weather at time of harvest. Therefore, the objective of this study was to determine the influence of cultivar and time of harvest on the nutrient content of freeze-dried mucilage. Knowledge of the nutrient components is necessary for recommendations in terms of the optimal cultivar and time of harvest to produce cactus pear mucilage.

## 2. Results and Discussion

### 2.1. Differences in the Nutrient Content Observed among the Four Cultivars

#### 2.1.1. Protein Content

**Figure 1:**
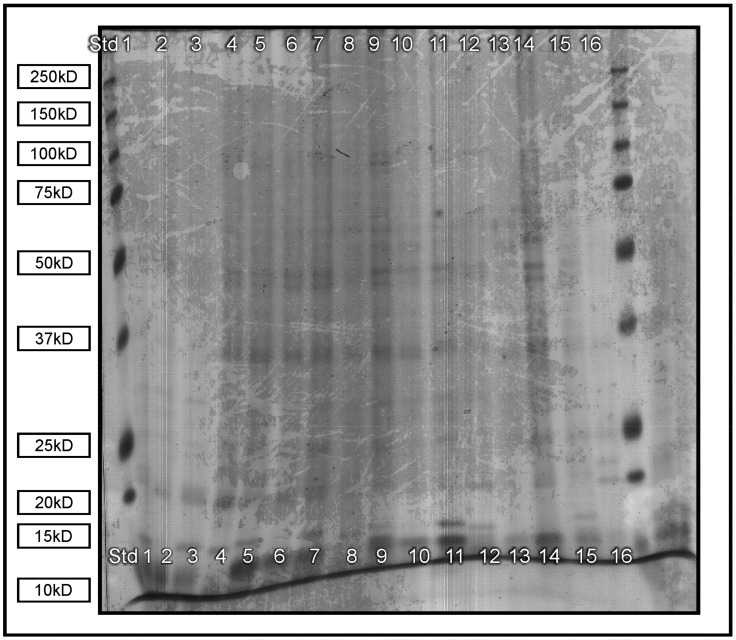
SDS-PAGE electrophoretogram image (February: 1 = Algerian, 2 = Morado, 3 = Gymno-Carpo, 4 = Robusta. May: 5 = Algerian, 6 = Morado, 7 = Gymno-Carpo, 8 = Robusta. June: 9 = Algerian, 10 = Morado, 11 = Gymno-Carpo, 12 = Robusta. August: 13 = Algerian, 14 = Morado, 15 = Gymno-Carpo, 16 = Robusta).

In the protein fingerprint determination SDS-PAGE electrophoretogram image (Figure 1), the most pronounced bands fell between 37 and 75 kD, which indicated the presence of prolamins (high in glutamine and proline). Other pronounced bands were observed at 10 kD (smallest proteins) in all samples except for Robusta (lanes 4, 8, 12 and 16), which indicated 2S albumin proteins present in the three *O. ficus-indica* cultivars, but not in *O. robusta*. 2S albumin functions as nutrient storage and as a protective protein in plants, but has been reported to be the cause of food allergies [11]. The prominence of glutamine [12,13], as well as albumin-like proteins [14], has been reported in cladodes.

The protein content of the three *O. ficus-indica* cultivars was not significantly different (2.7–3.2/100 g) (according to the statistical analysis *p* < 0.001) from each other, but was significantly lower than that of *O. robusta* (4.8/100 g; *p* < 0.001) [2]. The protein content of 6.8% reported [15] in mucilage was in the range of the mean protein content (3.4/100 g) determined in these mucilage samples [2]. The fraction of protein occurring in mucilage is small; however, it is consequential, as it grants mucilage the capacity to form emulsions by reducing the surface activity at the interphase. Mucilage has the ability to stabilize emulsions [16], act as an emulsifying agent [14], stabilizing agent [14], thickening agent [4,14] and serve as a fat replacement agent [17].

#### 2.1.2. Total Extractable Fat Content (EFC) and Fatty Acid Analysis

In Table 1, Robusta mucilage had higher EFC content than Gymno-Carpo and Algerian (*p* = 0.012). The most abundant fatty acid (Table 2) was the polyunsaturated linoleic acid (C18:2c9,12 (n-6)). It is rare for linoleic acid (Omega-6) to be predominant in plant materials [18]. Linoleic acid (Omega-6) is not synthesised in human cells, yet is indispensable in brain development, growth and cardiac health [19].

The other abundant fatty acid in mucilage powders was the monounsaturated oleic acid (C18:1c9) that is omnipresent in plant food sources and usually the most abundant fatty acid [18]. Together with saturated palmitic (C16:0) and stearic acid (C18:0), these four most abundant fatty acids made up 98.2% of the total fatty acids in the mucilage (Table 2). The SFA are deemed unhealthy, because the uncontrolled intake thereof is associated with high cholesterol, overweight, obesity and the potential cause of many chronic diseases [19,20]. Mucilage, however, contained less than 20% SFA. Foods rich in PUFA improve blood cholesterol levels, which can decrease the risk of cardiovascular diseases and diabetes [19]. The PUFA was 64.4% of the EFC (Table 2). The fat content of 0.4% reported in mucilage powders [15] was in the range of the mean EFC value (0.6/100 g) obtained in this study.

#### 2.1.3. Indigestible Carbohydrates

Low insoluble acid-detergent fibre and neutral-detergent fibre values (Table 1) indicated that mucilage was mostly pure soluble fibre (mostly mucilage and possibly pectin). Robusta had significantly higher neutral-detergent fibres than Algerian and Morado (Table 1). Thus, *O. robusta* mucilage would be the better choice when more insoluble fibre is required for a food product. The crude fibre value (0.1%) reported [15] in mucilage powders was lower than that obtained for *O. robusta* Robusta (0.7/100 g) and *O. ficus-indica* (0.2/100 g) mucilage in this study.

#### 2.1.4. Mineral Content

High levels of K (above 0.4 g/portion) are rare in many fresh foods [19], yet cactus pear cladodes have been reported to be a good natural source [21]. Robusta mucilage had significantly higher K content than Morado (*p* = 0.043), which was in the range of values reported [3] of between 1.6/100 g and 2.0/100 g in mucilage. Robusta had significantly lower Mg content (*p* < 0.001) than the *O. ficus-indica* cultivars (Table 3). The P levels detected in mucilage were unusually high for plant products (Table 3). 

#### 2.1.5. Calcium Oxalate Crystals

**Figure 2:**
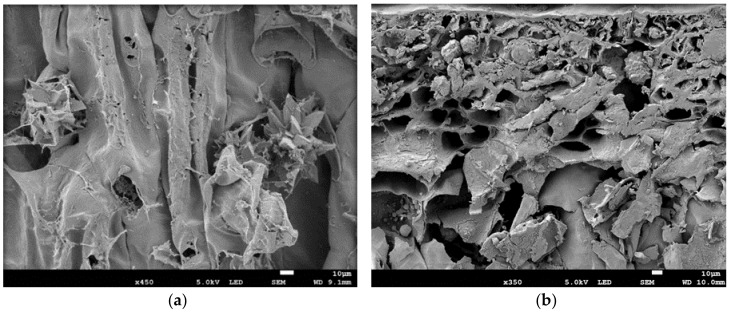
Fresh cladode parenchyma cells demonstrating different calcium oxalate crystal morphologies as (**a**) *Opuntia ficus-indica* (40–60 μm at ×450): three large whewellite stellate druses; (**b**) *Opuntia robusta* (±25 μm at ×550): three smaller crystals close to the epidermis.

Calcium oxalate crystals occur in Opuntioideae and have a negative effect on nutrition, as it binds with essential minerals such as calcium and cannot be absorbed in the human digestive system and could cause harm to the kidneys [22]. It was observed in this study that the calcium oxalate crystal morphology was different among cultivars as the crystals were bigger and more abundant in the fresh cladode tissues of the three *O. ficus-indica* (Algerian, Morado and Gymno-Carpo) cultivars than in *O. robusta*. To demonstrate, a typical calcium oxalate crystal observed in the *O. ficus-indica* cultivars is observed in Figure 2a. The typical whewellite stellate druses observed in *O. ficus-indica* tissue were large (ranged from 30 to 100 μm) and in abundance throughout the tissue. Similar calcium oxalate crystals in the form of whewellite or weddelite crystals have been reported [23,24]. However, in *O. robusta* tissue, the crystals were smaller (ranging from 6 µm to 35 µm), rounder, very scarce and mostly observed close to the epidermis (Figure 2b). The sparser occurrence of calcium oxalate crystals may be beneficial to consumer health, influence the lower Ca content of *O. robusta* cladodes (Table 3) and have an influence on the viscosity of the mucilage [5]. Nevertheless, no calcium oxalate crystals were observed in any of the dried mucilage samples, possibly due to the low solubility of calcium oxalate and the relatively low concentration of calcium oxalate found in soluble fibre [25]. It may be deduced that the calcium oxalate crystals did not co-extract when the mucilage was separated from the solids during the extraction process.

### 2.2. Differences in Nutrient Content Observed over the Six Months of Harvest

The cladode samples were harvested from the orchard situated in a semi-arid area where drastic changes in the climate takes place between summer and winter months. In summer (December to March), hot extreme daytime temperatures (above 30 °C) with frequent rainfall (above 35 mm) prevail, while the winters (June to September) are dry (below 18 mm), cold (−5 to 5 °C) and frost is severe [26]. Regarding the maintenance of the orchard, fertilization of the soil using 200 kg/ha KCl (50% K) took place at the end of April [27].

#### 2.2.1. Gross Energy and Digestible Carbohydrates

Gross energy levels increased significantly from February to May and again to August (Table 1). This increase was attributed to the slight, yet cumulative increases that were observed in the EFC (Table 1) as well as the increases in the carbohydrate and starch (Table 1) content over the same period. In fact, February and April mucilage powders had significantly lower digestible carbohydrate content levels compared to the other months (Table 1). The starch content dropped from February to April, before increasing every month to 7.69/100 g (significantly higher *p* = 0.032). Thus, the cladodes harnessed higher energy-containing components in late winter months. Lower gross energy content was reported in cladode flour (6.9 kJ/g) [28]. The mean digestible carbohydrate values obtained in this study of carbohydrates and starch was in the range of carbohydrates (60/100 g) and starch (7.63–13.9/100 g) values reported [29] in cladodes.

#### 2.2.2. Ash and Mineral Content

The ash values (Table 3) were significantly higher (*p* < 0.001) in February and April, compared to May to August. This decrease over months is the consequence of the decreases observed in most of the major and minor dietary elements (Table 3).

The values obtained in this study were lower than the ash values reported [15] (34.0%) for mucilage obtained from Ethiopia in February and March, which are warm (25–30 °C) and dry months.

The Ca content in February and April dropped significantly (*p* < 0.001) in May and never fully recovered until August. Similar to the ash values, the Ca values were higher in February and April (*p* < 0.001). Values of 3.4/100 g [21] and 3.6/100 g [30] compared well with mean Ca values reported in this study. Ca is bioavailable as the oxalate: calcium ratio is ≥1 [25] in cladodes. The consumption of young cladodes (nopalitos) increase Ca intake in the diet [13].

The content of Na in February was significantly higher (*p* = 0.034) than June, although it recovered somewhat in August (Table 3). The Mn content decreased significantly (*p* = 0.005) from February to April and showed some recovery in July. These values compared well with Mn values reported [13] in cladodes (30 and 290 mg/kg).

The Fe content also decreased significantly from February to April; however, a recovery took place in June and continued with significantly high (*p* = 0.002) levels in August. The Cu content decreased significantly from February to May and showed a slight trend towards recovery by August. The Zn content differed significantly between months, but no trends were apparent. The mean Zn content was higher than 0.8 mg/kg [12]. The fluctuations seen in this study were attributed to changes in climate (temperature, rainfall and frost), rather than cladode maturity as suggested by [13].

## 3. Materials and Methods

### 3.1. Sample Collection

Cladodes were obtained at the Waterkloof experimental cactus pear orchard near Bloemfontein, Free State, South Africa (29°10′53′′ S, 25°58′38′′ E). The area is 1348 m above sea level and receives 556 mm average annual rainfall. It is a summer rainfall, semi-arid region with winter frost, where the mid-day temperature is 26 to 29 °C in summer (January to April) and cools down during May to night temperatures close to 0 °C in winter (June, July) [26].

The orchid hosts forty *Opuntia ficus-indica* cultivars and two *Opuntia robusta* cultivars laid out in a randomized complete block design, with two replications per cultivar.

The mature cladodes from the current growth season (approximately six months old) of three *O. ficus-indica* cultivars (Algerian, Morado and Gymno-Carpo) and *O. robusta* (Robusta) were harvested for mucilage extraction, drying and evaluation. One cladode from each of the five plants, from both replications, were harvested in the post fruit-harvest stage, between 9:00 and 11:15 on 25 February, 15 April, 20 May, 10 June, 15 July and 12 August 2015; thus, from peak summer to late winter. The sampling of fresh cladode tissue for scanning electron microscopy (SEM) was done in February 2016. In order to standardize samples, young good-quality cladodes were collected from the middle and north side of the plant, at 1 m height. They were labelled, packaged and transported to the laboratory where they were refrigerated. Extraction of mucilage proceeded according to a patented method [31] that involved slicing, microwave cooking for 4 min (800 W), macerating, centrifuging at 8000 rpm for 15 min and freeze-drying for 72 h in a Perano freeze-drier at −60 °C. In order to prevent moisture absorption, one g calcium citrate was added to 20 g of mucilage powder (5%), before being packaged in sealed containers [32] and frozen (−18°C). The values resulting from subsequent analysis are presented on an “as is” basis (not “dry matter”), as it would be marketed and sold in its present form. Moisture content of freeze-dried powders ranged from 14.0 to 18.8/100 g (means 15.8/100 g) [33].

### 3.2. Methods

The Leco Automatic Calorimeter AC-500 series Oxygen Combustion Vessel (LECO Corporation, St. Joseph, MI, USA) was used to determine the gross energy as kJ/kg [34]. For Crude Protein (CP), nitrogen (N) was determined by thermal combustion using 0.6 g freeze-dried mucilage samples in a Leco Nitrogen analyser (FP-528, LECO Corporation, St. Joseph, MI, USA) [34]. Crude protein (CP/kg) was calculated by multiplying the N content by a factor of 6.25 [35].

For the separation of proteins, SDS-PAGE was performed on a C.B.S. DSG-200-02 instrument (C.B.S. Scientific Company, INC, Del Mar, CA, USA) at 16 °C. Freeze-dried mucilage samples of the four cultivars from February, May, June and August (0.2 g) were prepared [36]. February and August samples were used since it represented opposing growth stages and seasons of peak summer and late winter, while May and June samples represented the intermediary months. The samples (25 mL) were loaded into a gel matrix with the Precision plus protein all blue standard (Bio-Rad catalogue # 161-0373).

Total lipid content was quantitatively extracted [37], using chloroform and methanol in a ratio of 2:1. Total extractable fat content (EFC) was determined gravimetrically and expressed as % fat (*w*/*w*) per 100 g mucilage. Fatty acid analysis (FAME) (% of total fatty acids) was carried out using 10 mg of the total lipid extraction [38]. The FAME samples were identified by comparing relative peak retention times with standards obtained from SIGMA (189–19). Fatty acids were expressed as a percentage of the total of all fatty acids present in the sample. The total saturated fatty acids (SFA), total monounsaturated fatty acids (MUFA), total polyunsaturated fatty acids (PUFA) and PUFA/SFA ratios were calculated using the fatty acid data.

The total carbohydrate (%) content for mucilage was determined by subtracting the total moisture, protein, fats and ash contents [39]. Neutral-detergent fibre (NDF) and acid-detergent fibre (ADF) were determined using 1 g of freeze-dried mucilage samples [40,41]. This was expressed as g/kg. Starch in freeze-dried mucilage was determined according to the UV-method assay by Boehringer Mannheim/R-Biopharm Enzymatic Bioanalysis/Food Analysis Starch UV Method Cat No.:10,207,748,035 [42]. The starch content μmol/g was converted to g/100 g. 

The mineral analysis and ash determinations [43] were performed according to standard methods [44], using two grams of the freeze-dried powder of each sample in duplicate. Exchangeable Ca, Mg, K and Na (1 mol dm^−3^ NH_4_OAc at pH 7) and extractable Cu, Fe, Mn and Zn (DTPA solution) were determined by atomic absorption. Phosphorous (P) was determined colorimetrically (430 nm) according to the Vanado-Molybdate method [43].

Scanning electron microscopy (SEM) was conducted on both fresh cladode tissue and freeze-dried samples. The fresh cladode tissue was obtained from the four cultivars in February 2016. The freeze-dried mucilage powder samples from the previous year (2015) (as previously described) from the first month (February 2015) and the last month (August 2015) were used as the samples represent the opposing growth stages and seasons of peak summer and late winter. Freshly cut samples were prepared according to a method described by [45]. All samples were mounted on a metal stub, and a sputter coater was used to coat it with gold [45]. The samples were inspected using a JEOL scanning electron microscope (JSM-7800F Extreme-resolution Analytical Field Emission JEOL FE-SEM, JEOL USA, Inc., Peabody, MA, USA).

### 3.3. Statistical Methods

Analysis of variance (ANOVA) [46] was used to determine the effect of month of harvest and cultivar on the nutrient composition of freeze-dried mucilage samples. The Tukey-Kramer multiple comparison test (α = 0.05) was carried out to determine whether significant differences exist between treatment means [46].

## 4. Conclusions

The protein found in the mucilage powders may be too low to be nutritionally important, yet it provides mucilage with the ability to act as a functional ingredient. The change in climate from February to August significantly influenced the energy and mineral content. Mucilage extracted from cladodes in late winter months harnessed higher energy-containing components, while the Ca, Na, Mn, Fe and Cu contents dropped significantly after February. Optimal harvest time for mineral content was directly after the fruit harvest in February, as the energy content is the lowest and the dietary minerals the highest.

The protein fingerprint, EFC, fibre, K and Mg content and the shape and occurrence of calcium oxalate crystals were significantly different between the four cultivars. *O. robusta* powders had the highest content of crude protein, EFC and K, while the fresh cladode tissue had smaller and fewer calcium oxalate crystals. However, the three *O. ficus-indica* cultivars had the highest content of polyunsaturated fats (Linoleic and α-Linolenic acid). The selection of specific cultivars for their most beneficial components is recommended, depending on the purpose of mucilage powder application in functional food products. However, *O. robusta* Robusta harvested directly after the fruit harvest in February is recommended for the production of mucilage powders.

Cactus pear mucilage is a promising hydrocolloid that could replace unwanted ingredients in functional food products. The highest-quality mucilage was obtained from cladodes harvested in hot and dry conditions; therefore, it may present a rare opportunity for the commercial cultivation of cactus pears in arid and semi-arid areas.

## Figures and Tables

**Table 1:** Fat, gross energy, carbohydrate, starch and detergent fibre content of freeze-dried mucilage powders extracted from four cactus pear cultivars harvested from cladodes over six months.

		Extractable Fat Content	Gross Energy	Total Carbohydrates	Starch	Acid-Detergent Fibre	Neutral-Detergent Fibre
		g/100 g	kJ/g	g/100 g	g/100 g	g/kg	g/kg
Cultivar	Algerian	0.5 ± 0.2 ^a^	9.8 ± 0.9	62.4 ± 3.5	5.7 ± 1.1	0.9 ± 0.8	0.7 ± 0.6 ^a^
	Morado	0.6 ± 0.2 ^ab^	10.3 ± 1.1	62.8 ± 3.5	5.5 ± 0.1	1.1 ± 0.1	1.4 ± 0.1 ^a^
	Gymno-Carpo	0.4 ± 0.2 ^a^	10.1 ± 0.1	62.6 ± 3.4	6.1 ± 0.9	1.5 ± 1.5	1.7 ± 2.1 ^ab^
	Robusta	0.9 ± 0.3 ^b^	10.1 ± 0.1	62.1 ± 5.2	6.5 ± 1.9	2.3 ± 1.9	4.5 ± 3.1 ^b^
	Significance level	(*p* = 0.012)	(*p* = 0.332)	(*p* = 0.989)	(*p* < 0.586)	(*p* = 0.321)	(*p* = 0.014)
Month	February	0.5 ± 0.2	9.1 ± 0.3 ^a^	59.1 ± 2.7 ^a^	5.4 ± 0.3 ^ab^	1.4 ± 1.1	1.6 ± 0.4
	April	0.4 ± 0.2	9.2 ± 0.6 ^a^	56.6 ± 0.6 ^a^	5.1 ± 1.1 ^a^	0.5 ± 0.4	0.6 ± 0.5
	May	0.7 ± 0.2	10.3 ± 0.5 ^b^	63.6 ± 0.9 ^b^	5.5 ± 0.9 ^ab^	1.8 ± 1.6	3.3 ± 3.1
	June	0.7 ± 0.2	10.6 ± 0.3 ^bc^	65.8 ± 1.0 ^b^	5.7 ± 0.8 ^ab^	2.4 ± 0.3	2.0 ± 1.8
	**July**	0.6 ± 0.4	10.7 ± 0.6 ^bc^	64.9 ± 0.6 ^b^	6.2 ± 0.9 ^ab^	1.2 ± 2.3	2.7 ± 4.5
	**August**	0.8 ± 0.4	11.6 ± 0.5 ^c^	64.9 ± 1.9 ^b^	7.7 ± 1.7 ^b^	1.4 ± 1.4	2.4 ± 1.5
	**Significance level**	(*p* = 0.359)	(*p* < 0.001)	(*p* < 0.001)	(*p* = 0.032)	(*p* = 0.523)	(*p* = 0.694)
	**Means**	0.6 ± 0.2	10.2 ± 0.1	62.5 ± 3.7	5.6 ± 0.9	1.4 ± 0.7	2.1 ± 0.1

*Opuntia ficus-indica* (Algerian, Morado and Gymno-Carpo), *Opuntia robusta* (Robusta); Cultivar means with different superscripts ^a, b, c^ in the same column differ significantly (*p* < 0.05) and represent the means of data obtained over the six harvest months; Month means with different superscripts ^a, b, c^ in the same column differ significantly (*p* < 0.05) and represent the means of data obtained from the four cultivars.

**Table 2:** Fatty acid content (% of total extractable fat) and ratios of freeze-dried mucilage powders extracted from four cactus pear cultivars harvested from cladodes over six months (February, April, May, June, July and August).

	Fatty Acids	Abbreviation	Algerian	Morado	Gymno-Carpo	Robusta	Mean	Significance Level
			% of Total Fatty Acids					
Individual fatty Acids:	Myristic	C14:0	0.1 ± 0.03	0.1 ± 0.01	0.1 ± 0.02	0.1 ± 0.01	0.1 ± 0.01	*p* = 0.614
	Palmitic	C16:0	14.1 ± 1.8	13.8 ± 1.2	12.8 ± 1.5	15.2 ± 1.0	14.0 ± 0.5	*p* = 0.094
	Palmitoleic	C16:1c9	0.7 ± 0.04	0.7 ± 0.1	0.6 ± 0.2	0.6 ± 0.1	0.7 ± 0.05	*p* = 0.142
	Margaric	C17:0	0.02 ± 0.001	0.02 ± 0.001	0.02 ± 0.001	0.02 ± 0.001	0.02 ± 0.001	*p* = 0.103
	Heptadecenoic	C17:1c10	0.04 ± 0.01	0.1 ± 0.04	0.1 ± 0.1	0.1 ± 0.02	0.1 ± 0.02	*p* = 0.59
	Stearic acid	C18:0	3.1 ± 0.1	2.9 ± 0.4	2.9 ± 0.3	2.9 ± 0.4	3.0 ± 0.4	*p* = 0.823
	Oleic	C18:1c9	15.6 ± 4.5 ^a^	20.1 ± 4.1 ^ab^	16.1 ± 5.4 ^ab^	23.5 ± 4.2 ^b^	19.1 ± 1.8	*p* = 0.023
	Linoleic	C18:2c9,12 (n−6)	65.3 ± 5.2 ^ab^	60.6 ± 5.0 ^ab^	66.5 ± 6.8 ^b^	56.2 ± 5.2 ^a^	62.1 ± 2.0	*p* = 0.018
	Arachidic	C20:0	0.1 ± 0.04 ^a^	0.2 ± 0.1 ^a^	0.1 ± 0.04 ^a^	0.5 ± 0.12 ^b^	0.2 ± 0.1	*p* < 0.001
	Eicosenoic	C20:1c11	0.2 ± 0.02	0.2 ± 0.02	0.2 ± 0.02	0.2 ± 0.03	0.2 ± 0.01	*p* = 0.335
	α-Linolenic	C18:3c9,12,15 (n-3)	0.2 ± 0.1 ^a^	0.2 ± 0.1 ^a^	0.1 ± 0.1 ^a^	0.3 ± 0.04 ^b^	0.2 ± 0.1	*p* = 0.018
	Behenic	C22:0	0.1 ± 0.03 ^a^	0.2 ± 0.1 ^ab^	0.2 ± 0.1 ^ab^	0.2 ± 0.04 ^b^	0.2 ± 0.02	*p* = 0.041
	Eicosatrienoic	C20:3c8,11,14 (n-6)	0.1 ± 0.1 ^a^	0.1 ± 0.1 ^a^	0.1 ± 0.1 ^a^	0.4 ± 0.2 ^b^	0.2 ± 0.1	*p* < 0.001
	Lignoceric	C24:0	0.03 ± 0.02	0.04 ± 0.02	0.03 ± 0.02	0.1 ± 0.03	0.04 ± 0.02	*p* = 0.163
Fatty Acid Ratios:	Saturated Fatty Acids		17.7 ± 2.7	17.3 ± 1.3	16.3 ± 1.5	18.8 ± 1.2	17.5 ± 0.5	*p* = 0.117
	Monounsaturated		16.8 ± 4.5 ^a^	21.9 ± 4.1 ^ab^	17.0 ± 5.4 ^a^	24.3 ± 4.1 ^b^	20.0 ± 1.2	*p* = 0.023
	Polyunsaturated		65.5 ± 5.1 ^ab^	60.9 ± 4.5 ^ab^	66.8 ± 6.7 ^b^	56.9 ± 5.2 ^a^	64.4 ± 1.9	*p* = 0.022
	PUFA:SFA Ratio		3.8 ± 0.8 ^ab^	3.6 ± 0.6 ^ab^	4.2 ± 0.7 ^b^	3.0 ± 0.4 ^a^	3.6 ± 0.2	*p* = 0.044

*Opuntia ficus-indica* (Algerian, Morado and Gymno-Carpo), *Opuntia robusta* (Robusta). Means with different superscripts ^a, b, c^ in the same row differ significantly *p* < 0.05. and represent the means of data obtained over the six harvest months.

**Table 3:** Micronutrient content of freeze-dried mucilage powders extracted from four cactus pear cultivars harvested from cladodes over six months.

		Ash	Ca	K	Mg	P	Na	Mn	Fe	Cu	Zn
		g/100 g	g/100 g	g/100 g	g/100 g	mg/kg	mg/kg	mg/kg	mg/kg	mg/kg	mg/kg
Cultivar	Algerian	18.8 ± 2.5	3.3 ± 0.9	2.9 ± 0.7 ^ab^	2.6 ± 0.3 ^b^	113.8 ± 24.1	86.3 ± 34.6	190.9 ± 30.1	32.3 ± 25.3	6.2 ± 1.0	23.7 ± 4.6
	Morado	17.1 ± 2.0	3.0 ± 0.9	2.3 ± 0.5 ^a^	2.6 ± 0.2 ^b^	93.9 ± 11.3	129.3 ± 50.1	185.8 ± 118.7	18.3 ± 18.0	5.7 ± 0.7	25.7 ± 4.2
	Gymno-Carpo	18.1 ± 2.5	3.0 ± 0.8	2.6 ± 1.0 ^ab^	2.7 ± 0.3 ^b^	100.2 ± 26.7	114.3 ± 46.4	220.7 ± 88.1	15.7 ± 18.8	5.4 ± 1.1	24.3 ± 3.0
	Robusta	16.8 ± 3.4	2.3 ± 1.2	3.3 ± 0.1 ^b^	2.0 ± 0.1 ^a^	129.9 ± 132.0	143.2 ± 72.6	156.2 ± 84.1	22.2 ± 23.7	4.9 ± 1.2	24.5 ± 6.4
	Significance level	(*p* = 0.545)	(*p* = 0.854)	(*p* = 0.043)	(*p* < 0.001)	(*p* = 0.808)	(*p* = 0.311)	(*p* = 0.647)	(*p* = 0.572)	(*p* = 0.235)	(*p* = 0.904)
Month	February	21.0 ± 1.1 ^b^	3.5 ± 0.9 ^bc^	3.7 ± 0.8	2.8 ± 0.4	125.2 ± 26.3	170.0 ± 18.7 ^b^	302.5 ± 82.1 ^b^	31.6 ± 4.8 ^ab^	7.1 ± 0.8 ^b^	27.8 ± 3.3 ^b^
	April	20.1 ± 1.1 ^b^	4.6 ± 0.4 ^c^	2.6 ± 1.0	2.4 ± 0.2	103.0 ± 18.1	133.1 ± 92.9 ^ab^	136.9 ± 16.4 ^a^	3.9 ± 1.3 ^a^	5.6 ± 0.5 ^ab^	26.5 ± 5.1 ^ab^
	May	17.0 ± 0.7 ^a^	2.1 ± 0.4 ^a^	2.8 ± 0.5	2.5 ± 0.4	85.9 ± 14.5	79.8 ± 14.5 ^ab^	136.4 ± 50.4 ^a^	2.6 ± 1.3 ^a^	5.0 ± 0.4 ^a^	28.3 ± 4.3 ^b^
	June	16.1 ± 1.5 ^a^	2.5 ± 0.5 ^ab^	2.4 ± 0.5	2.5 ± 0.4	166.2 ± 156.1	70.5 ± 20.5 ^a^	129.9 ± 53.2 ^a^	16.9 ± 28.1 ^ab^	4.9 ± 1.4 ^a^	22.3 ± 1.7 ^ab^
	July	15.6 ± 1.2 ^a^	2.6 ± 0.4 ^ab^	2.6 ± 0.4	2.3 ± 0.3	88.7 ± 20.6	113.0 ± 37.9 ^ab^	222.3 ± 65.0 ^ab^	28.8 ± 20.9 ^ab^	5.3 ± 0.7 ^a^	20.5 ± 3.3 ^a^
	August	15.6 ± 1.4 ^a^	2.75 ± 0.5 ^ab^	2.5 ± 0.2	2.3 ± 0.4	87.8 ± 16.8	143.3 ± 38.4 ^ab^	202.4 ± 80.2 ^ab^	48.9 ± 9.0 ^b^	5.4 ± 0.9 ^ab^	22.0 ± 3.0 ^ab^
	Significance level	(*p* < 0.001)	(*p* < 0.001)	(*p* = 0.090)	(*p* = 0.463)	(*p* = 0.498)	(*p* = 0.034)	(*p* = 0.005)	(*p* = 0.002)	(*p* = 0.015)	(*p* = 0.023)
	Means	17.7 ± 2.3	3.0 ± 0.9	2.8 ± 0.5	2.5 ± 0.2	109.5 ± 31.5	118.3 ± 38.3	188.4 ± 68.0	22.1 ± 17.8	5.5 ± 0.8	24.5 ± 3.34
	% Ash		−	−	−	−	−	0.01	0.002	N/A	0.002

*Opuntia ficus-indica* (Algerian, Morado and Gymno-Carpo), *Opuntia robusta* (Robusta). Cultivar means with different superscripts ^a, b, c^ in the same column differ significantly (*p* < 0.05) and represent the means of data obtained over the six harvest months. Month means with different superscripts ^a, b, c^ in the same column differ significantly (*p* < 0.05) and represent the means of data obtained from the four cultivars.

## Abbreviations

CAM	Crassulacean acid metabolism
EFC	Extractable fat content
MUFA	Monounsaturated fatty acids
PUFA	Polyunsaturated fatty acids
SFA	Saturated fatty acids
